# Smart Rumble Strip System to Prevent Over-Height Vehicle Collisions

**DOI:** 10.3390/s24196191

**Published:** 2024-09-25

**Authors:** Ricky W. K. Chan

**Affiliations:** School of Engineering, STEM College, RMIT University, Melbourne, VIC 3000, Australia; ricky.chan@rmit.edu.au

**Keywords:** over-height vehicles, collisions, smart structures, rumble strips

## Abstract

Collisions of over-height vehicles with low clearance bridges is commonly encountered worldwide. They have caused damage to bridge structures, interruption to traffic, injuries or even fatalities to road users. To mitigate such risks, passive systems that involve warning gantries, flashing lights and illuminated signage are commonly installed. Semi-active systems using laser- or infrared-based detection systems in conjunction with visual warnings have been implemented. Nevertheless, some drivers ignore these visual warnings and collisions continue to occur. This paper presents a novel concept for a collision prevention system, which makes use of a series of sensor-activated, motorized rumble strips. These rumble strips span across a certain distance ahead of a low clearance bridge. When an over-height vehicle is detected, a mechanism is triggered which elevates the rumble strips. The noise and vibrations produce a vigorous alert to the offending driver. They also increase effective friction of the road surface, thus assisting to slow down the vehicle and shorten the stopping distance. The strips will be lowered after a certain time has elapsed, thus minimizing their effects on other vehicles. This article presents a conceptual framework and quantifies the vibration and noise caused by rumble strips in road tests. Road tests indicated that the vibration level typically exceeded 1 g and noise level reached approximately 90 dB in the cabin of a 3.5-ton truck. Fabrication of a proof-of-concept mechanized rumble strip model was presented and verified in an outdoor environment. The circuitry and mechanical design, and requirements in actual implementation, are discussed. The proposed event-triggered rumble strip system could significantly mitigate over-height vehicle collisions that cause major disruptions and injuries worldwide. Further works, including a comprehensive road test involving various types of vehicles, are envisaged.

## 1. Introduction

**Figure 1:**
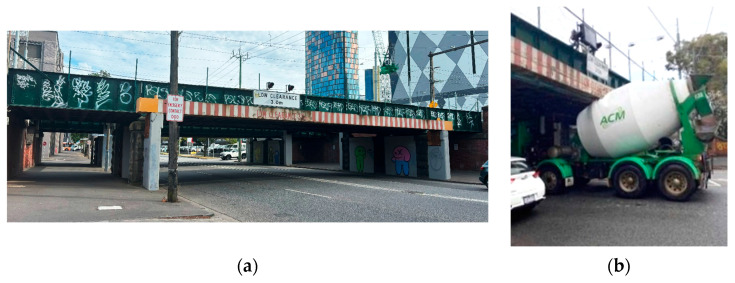
(**a**) The Montague Street bridge, Melbourne, Australia with 3.0 m clearance, (**b**) a collision event in November 2022.

Over-height vehicles striking low clearance bridges, tunnel openings or overhead wires is a global problem that can result in disastrous consequences. In low-speed collisions, strikes result in minor damage to the bridge structure and the vehicle involved, while in high-speed collisions, significant structural damage, injuries or fatalities to road users or pedestrians nearby could be caused. Damage to bridge structures is expensive to repair, disruptive to traffic flow and will lead to economic losses and inconvenience to commuters. The allocation of time and resources, such as emergency services, traffic management and incident response teams in the event of a collision also takes its toll not only financially but also because the time and effort of these services could be spared to attend to other incidents throughout the community. In addition, depending on the circumstances, a collision may result in legal, insurance and liability issues that may fall on the driver of the offending vehicle and transportation authorities. In 2013, an over-height vehicle struck the I-5 Skagit River bridge in Washington, USA and caused the bridge to collapse. Two vehicles with three people fell into the river [1]. In 2016, an over-height truck collided with a concrete pedestrian bridge on the M20 Motorway, southeast of London, UK, and caused the entire concrete bridge section to collapse onto the motorway [2]. In Melbourne, Australia, the city where the author resides, the 3.0 m clearance Montague Street bridge (Figure 1), was collided with 106 times between 2012 and 2016 [3]. As shown in Figure 1a, warning signs and flashing lights have been installed, yet collisions continue to occur. In February 2016, a 3.8 m high passenger coach collided with the bridge. Its roof was torn off and the coach wedged under the bridge. The incident caused major injuries to three passengers of the bus [4]. Figure 1b shows the same bridge was struck by a concrete truck in November 2022. A study conducted by the state of Maryland in the USA indicated that there were 1496 bridges that are prone to impact due to over-height vehicles statewide, and 20% of them have been struck [5]. In the event of an over-height vehicle carrying dangerous goods, such as flammable liquids, catastrophic consequences can be expected.

### 1.1. Review of Risk Mitigation Methods against Over-Height Vehicle Striking

**Figure 2:**
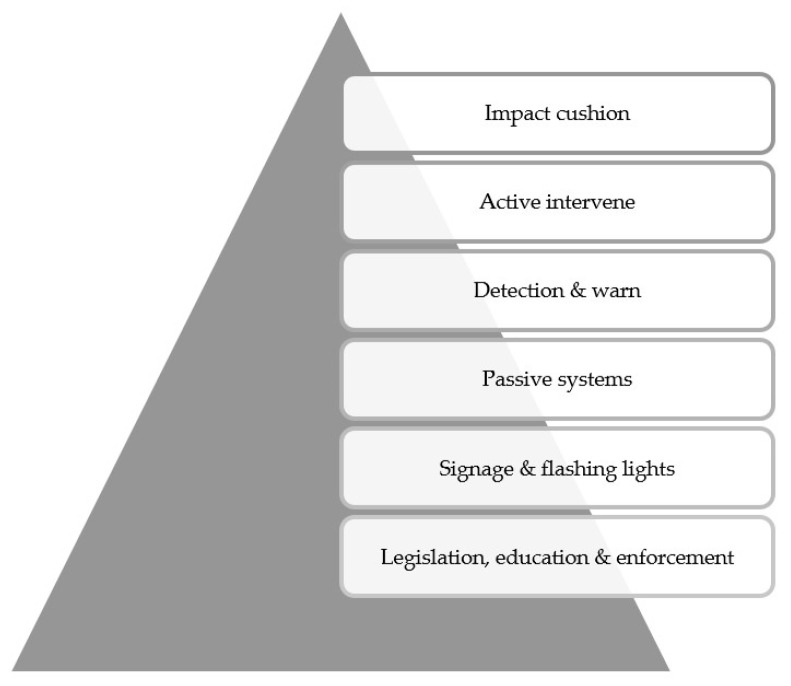
Hierarchy of over-height vehicle impact risk mitigation [8].

As in all other road hazards, the fundamental risk mitigation is realized by engineering, driver education, legislation, and enforcement. In most jurisdictions, truck drivers are required to pass heavy vehicle license assessments that comprise both knowledge tests and road tests. Information about the locations and mapping of low clearance structures are, by law, required to be in over-height vehicles in the state of Victoria, Australia. Over-height permits, the enforcement of fines, and displaying the height of a vehicle inside the cabin are common measures to alert drivers. Drivers have the responsibility to reroute their vehicles to avoid low clearance bridges. Osegueda et al. [6] presented a GIS-based rerouting method for over-height vehicles. While the legislation side is beyond the scope of this paper, some experience from the UK has been summarized by Nguyen [7]. Common occurrences of collisions indicate that mere legislation, driver education and enforcement are insufficient to protect road users. Figure 2 shows the state-of-practice hierarchy of risk mitigation techniques that have been developed. Six levels of risk mitigation measures are presented in the order of level of intervention. On the top of the hierarchy diagram, impact cushions are the last resort in the risk mitigation of collisions. They attempt to minimize damage to both the vehicle and the bridge structure.

### 1.2. Warning Signs and Passive Warning Systems

**Figure 3:**
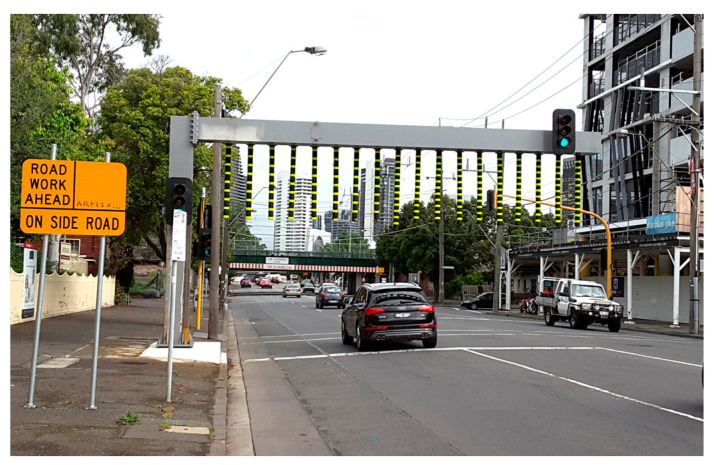
Passive system—warning gantry ahead of a low clearance bridge on Montague Street, Melbourne.

The most common risk mitigation is warning signs and flashing lights. In Australia, sign designation R6-11 and R6-12 of Australian Standard AS1742.1 [9] specify the requirements of such warning signs to warn drivers of a low clearance hazard ahead. An example is shown in Figure 1a in which a 3.0 m clearance sign is displayed on the Montague bridge located in the south side of the central business district of Melbourne. This method assumes drivers have knowledge of the height of their operating vehicle and react accordingly. This is, in fact, a common assumption that the majority of drivers are cautious, responsible and obey road signs, such as traffic lights and stop signs. However, traffic lights are far more common, and drivers are typically trained to respond accordingly. Drivers may ignore low clearance signage and flashing lights, as they normally pay attention to traffic conditions instead of overhead hazards. It is surprisingly common for drivers to ignore clearance signs. A study utilized yellow and black bridge markings to enhance the outline of the clearance, and this was found to be effective. Several bridge marking strategies to attract drivers’ attention were summarized in [10]. Passive systems are also widely used to warn drivers of potential hazards. Warning gantries with hanging chains or rubber flaps are commonly adopted; they provide a soft hit that does not cause damage to vehicles but creates a noise to alert drivers prior to collision with the bridge. Figure 3 shows a passive soft hit system, a warning gantry installed ahead of the Montague Street bridge, South Melbourne. The soft hanging bars on this warning gantry illuminate at night to enhance visibility.

### 1.3. Over-Height Detection and Warning (OHD-W) Systems

**Figure 4:**
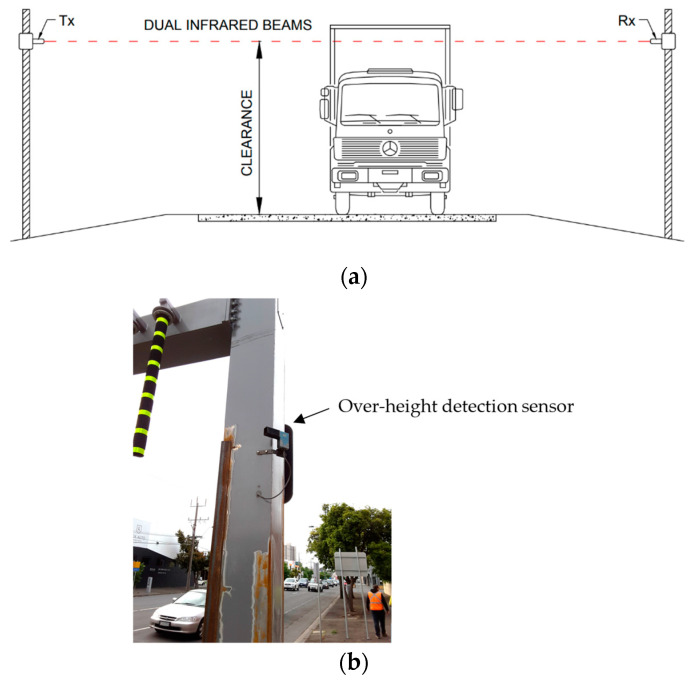
A dual infrared beam over-height detection system: (**a**) schematic diagram and (**b**) sensor installed on Montague Street, Melbourne.

In the 1980s, over-height vehicle detection was identified as a need to reduce the risk of collisions to truss bridges in the Mississippi river with trucks carrying timber logs. These trucks facilitated important economic activities [11]. Most OHD-W systems use optoelectronics methods such as laser or infrared beams. A schematic diagram is shown in Figure 4a. These systems usually consist of a transmitter (Tx) and receiver (Rx) that are located on opposite sides of the road. To detect the direction of travel, the transmitter emits two infrared beams. Once the beam is broken, an over-height event is triggered. To reject events caused by birds or flying debris, a dual-beam system can be used, in which an event is not triggered unless both beams are broken within a pre-determined time interval. The dual-beam arrangement may also be used to determine the direction of traffic and its speed. In the USA, OHD-W systems are becoming popular. In a recent study conducted by Purdue University, OHD-W systems were inexpensive to install and operate, and required minimal maintenance [12]. This suggests that they are an effective method for decreasing over-height vehicle accidents. On the other hand, microwave radar technology has also been studied to identify vehicle heights [13]. Other strategies to detect over-height vehicles include combinations of horizontal lasers and vertical ultrasonic sensors [14], and computer vision-based technology has been reported in which vehicle height is estimated using line detection and blob tracking techniques over recorded video images [15].

### 1.4. Broadcasting and Vehicle Intervention

In the research into intelligent transport systems, a research institute in Australia, NICTA, developed a technology called “TruckOn” [16]. In this proposed technology, a “black box” is installed in a vehicle, which communicates wirelessly with a control center. Once an offending vehicle is detected by a beam break detector, a warning message is broadcast into the offending vehicle’s cabin (not to other vehicles). The black box monitors the driver’s behavior and alters the warning message accordingly. If the warning is ignored, the black box may apply the brakes and stop the offending vehicle. It is an advanced information and communication technology, which involves the wireless communication of vehicles with roadside infrastructure. However, concerns over this technology, such as the capital investment into roadside communication infrastructure and the installation of a black box in every heavy vehicle, may hinder the adaptability of this technology. In addition, particularly because the vehicle could be controlled remotely, concerns over cyber-security, software reliability and legal issues have been raised.

### 1.5. Impact Cushions and Structural Health Monitoring

As a last resort, impact cushions can be installed on bridges to reduce damage in the event of collision. These impact cushions dissipate the impact energy and effectively reduce the damage to a bridge structure, which is difficult and costly to repair. The cushion can be installed on existing bridges, and damaged cushions can be readily replaced. Thus, they are a cost-effective way to mitigate collision risks. An impact cushion made of aluminum honeycomb panels, which was designed to reduce impact damage on a concrete girder, was developed in the USA [17,18]. Baral et al. [19] also described a strain hardening concrete mixture that has impact resistance characteristics.

On the other hand, the need for road authorities to receive information in the event of a collision is crucial in terms of immediate damage evaluation, public safety and traffic management. Sensor networks based on fiber optics [20] and piezoelectric transducers [21] were proposed to detect collisions. The sensor networks also provide structural performance data through structural dynamic principles, which are the underlying principles of structural health monitoring. It was also proposed that a collision event could trigger a camera to take photographic records of offending vehicles [21]. Das et al. [22] provided a comprehensive state-of-the-art review on strategies for the protection and repair of bridges vulnerable to over-height vehicle collisions as well as prevention strategies.

### 1.6. Objectives of This Work

Existing OHD-W systems use visual and, sometimes, audible alerts only to alert drivers, which have been found to be ineffective on many occasions. Bridges with low clearance, such as the Montague Street bridge in Melbourne, have been struck repeatedly despite the warning signs, flashing lights and soft hit bars that have been installed. This clearly indicates that drivers have been ignoring these conventional warnings. To address this engineering problem, this paper describes a novel, active warning technique to prevent over-height vehicles collision. The proposed system combines the optoelectronics method for over-height detection and a mechanized rumble strip system. Once an event is detected, it triggers a mechanism that deploys a series of rumble strips. The rumble strips produce severe noise and vibrations to alert the offending driver of an upcoming hazard. The strips also effectively increase road friction and assist in decelerating the offending vehicle, thus reducing the damage of impact. The concept was first proposed by the author in a conference article entitled “Over-height Collision Prevention System with Smart Rumble Strips” [8], which was presented at the Austroad Bridge Conference in Melbourne, Australia in 2017. This article presents a revised design and elaborates on several areas. It first reviews the state-of-practice risk mitigation measures and technologies, followed by a description of the proposed system. A proof-of-concept model testbed is presented. Lastly, key features and requirements for real-life application are discussed. The proposed system would provide a safety net to address the issue of accidents occurring due to low clearance bridges.

## 2. Conceptual Framework of the Smart Rumble Strips

Transverse rumble strips are used worldwide to alert drivers of potential hazards and to slow down traffic. Bituminous, indented rumble strips are commonly applied on freeway shoulders (or breakdown lanes) to alert drivers of lane departure. These produce a humming noise and tactile vibrations to alert drivers; yet, the vibration level is not high enough to alter the path of vehicles. Rumble strips are also used in leveled railway crossings to alert drivers to decelerate. Thermoplastic rumble strips are available as a means of post-paving installation—rumble strip units are screwed into the road pavement. The effectiveness of rumble strips has been comprehensively studied [23,24]. On the other hand, existing OHD-W technologies mainly use warning signs, flashing lights and audible bells to warn offending drivers and clearly fail to attract attention on many occasions. This work presents a novel approach—smart, mechanized rumble strips that are activated by the detection of an over-height vehicle.

**Figure 5:**
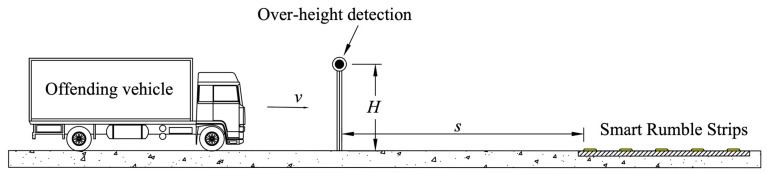
Proposed smart rumble strip system.

Figure 5 shows a schematic diagram of the concept. *H* denotes the height at which the detection system—for example, an infrared beam break system—is installed. When an offending vehicle is detected, a mechanical system is activated to raise the smart rumble strips. *s* denotes the distance between the point of detection and the first rumble strip. The distance *s* can be determined by the response time of the mechanical system and the design speed of the vehicle *v*.

### Theoretical Framework

**Figure 6:**
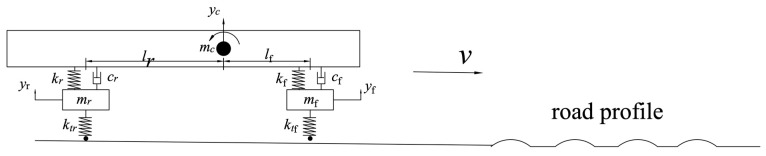
Vehicle dynamics represented by 4-DOF half car model.

The proposed smart rumble strip system utilizes the generated vibrations and noise in the cabin to alert drivers. Vehicle vibrations due to road surface profile are a classical field of study in vehicle dynamics. Differential equations to describe each degree of freedom have been widely studied, and mathematical models of various sophistications have been widely studied, ranging from a simple quarter car model [25,26], to a half car model [27,28], and a three-dimensional full car model [29]. This section presents a vehicle passing over a road surface profile with a 4-degree-of-freedom half car model, as shown in Figure 6. The equations of motion describe the vertical motion (bounce) of the chassis (Equation (1)), the rotation about the lateral axis (Equation (2)), and the front and rear suspension vertical motion (Equations (3) and (4), respectively). The road profile is a time-domain signal input to the system. This model includes the vehicle body and two suspension systems, one for the front and one for the rear axle. The suspension systems are characterized by the spring stiffness and damping constant.

(1) $$m_{c}{\ddot{y}}_{c}+c_{f}\left({\dot{y}}_{c}+l_{f}\dot{\theta }-{\dot{y}}_{f}\right)+k_{f}\left(y_{c}+l_{f}\theta -y_{f}\right)+c_{r}\left({\dot{y}}_{c}-l_{f}\dot{\theta }-{\dot{y}}_{r}\right)+k_{r}\left(y_{c}-l_{f}\theta -y_{r}\right)=0$$

(2) $$I\ddot{\theta }+l_{f}c_{f}\left({\dot{y}}_{c}+l_{f}\dot{\theta }-{\dot{y}}_{f}\right)+l_{f}k_{f}\left(y_{c}+l_{f}\theta -y_{f}\right)-l_{r}c_{r}\left({\dot{y}}_{c}-l_{r}\dot{\theta }-{\dot{y}}_{r}\right)-l_{r}k_{r}\left(y_{c}-l_{r}\theta -y_{r}\right)=0$$

(3) $$m_{f}{\ddot{y}}_{f}{-k}_{f}\left(y_{c}+l_{f}\theta -y_{f}\right)-c_{f}\left({\dot{y}}_{c}+l_{f}\dot{\theta }-{\dot{y}}_{f}\right)+k_{tf}\left(y_{f}-y_{rf}\right)=0$$

(4) $$m_{r}{\ddot{y}}_{r}{-k}_{r}\left(y_{c}-l_{r}\theta -y_{r}\right)-c_{r}\left({\dot{y}}_{c}+l_{r}\dot{\theta }-{\dot{y}}_{f}\right)+k_{tr}\left(y_{r}-y_{rr}\right)=0$$

where

- *m_c_*: Mass of the vehicle chassis;
- *m_f_*, *m_r_*: Mass of the front and rear axle;
- *I*: Moment of inertia of the vehicle body about the center of gravity;
- *k_f_*, *k_r_*, *k_tr_*, *k_tf_*: Spring constants of the front suspension, rear suspension, front tire and rear tire, respectively;
- *c_f_*, *c_r_*: Damping coefficients of the front and rear suspensions;
- *l_f_*, *l_r_*: Distance from the CG to the front and rear axles;
- *y_c_*, *y_f_*, *y_r_*: Vertical displacement of the vehicle body’s center of gravity and the front and rear axles;
- *θ*: Pitch angle of the vehicle body;
- *y_rf_*, *y_rr_*: Road input at front and rear axles.

To solve these equations numerically, we can convert the governing equations into a state–space representation. Define the state vector **x** as follows:

(5) $$x={\left[y_{c},\theta ,y_{f},y_{r},{\dot{y}}_{c},\dot{\theta },{\dot{y}}_{f},{\dot{y}}_{r}\right]}^{T}$$

The state–space equations are:

(6) $$\dot{x}=Ax+Bu$$

(7) y=Cx+Du

where the **A**, **B**, **C** and **D** matrices are derived from the equations of motion, and **u** is the input vector that consists of the following road profile:

(8) $$u={\left[y_{rf},y_{rr}\right]}^{T}$$

(9) $$A=\left[\begin{array}{cc} 0 & I\\ K/m & C/m \end{array}\right]$$

(10) $$\frac{K}{m}=\left[\begin{array}{cccc} -\frac{k_{sf}+k_{sr}}{m_{c}} & \frac{k_{sf}L_{f}+k_{sr}L_{r}}{m_{c}} & \frac{k_{sf}}{m_{c}} & \frac{k_{sr}}{m_{c}}\\ \frac{k_{sf}L_{f}-k_{sr}L_{r}}{I_{c}} & -\frac{k_{sf}L_{f}^{2}+k_{sr}L_{r}^{2}}{I_{c}} & -\frac{k_{sf}L_{f}}{I_{c}} & \frac{k_{sr}L_{r}}{I_{c}}\\ \frac{k_{sf}}{m_{f}} & -\frac{k_{sf}L_{f}}{m_{f}} & -\frac{k_{tf}+k_{sf}}{m_{f}} & 0\\ \frac{k_{sr}}{m_{r}} & \frac{k_{sr}L_{r}}{m_{r}} & 0 & -\frac{k_{tr}+k_{sr}}{m_{r}} \end{array}\right]$$

(11) $$\frac{C}{m}=\left[\begin{array}{cccc} -\frac{c_{sf}+c_{sr}}{m_{c}} & \frac{c_{sf}L_{f}+c_{sr}L_{r}}{m_{c}} & \frac{c_{sf}}{m_{c}} & \frac{c_{sr}}{m_{c}}\\ \frac{c_{sf}L_{f}-c_{sr}L_{r}}{I_{c}} & -\frac{c_{sf}L_{f}^{2}+c_{sr}L_{r}^{2}}{I_{c}} & -\frac{c_{sf}L_{f}}{I_{c}} & \frac{c_{sr}L_{r}}{I_{c}}\\ \frac{c_{sf}}{m_{f}} & -\frac{c_{sf}L_{f}}{m_{f}} & -\frac{c_{sf}}{m_{f}} & 0\\ \frac{c_{sr}}{m_{r}} & \frac{c_{sr}L_{r}}{m_{r}} & 0 & -\frac{c_{sr}}{m_{r}} \end{array}\right]$$

(12) $$B=\left[\begin{array}{cc} 0 & 0\\ 0 & 0\\ 0 & 0\\ 0 & 0\\ \frac{k_{tf}}{m_{c}} & \frac{k_{tr}}{m_{c}}\\ \frac{k_{tf}L_{f}}{I_{c}} & -\frac{k_{tr}L_{r}}{I_{c}}\\ -\frac{k_{tf}}{m_{f}} & 0\\ 0 & -\frac{k_{tr}}{m_{r}} \end{array}\right]$$

**C** is the output matrix and **D** is the direct transmission matrix, where C=I, **D = 0** and **I** is the identity matrix.

**Figure 7:**
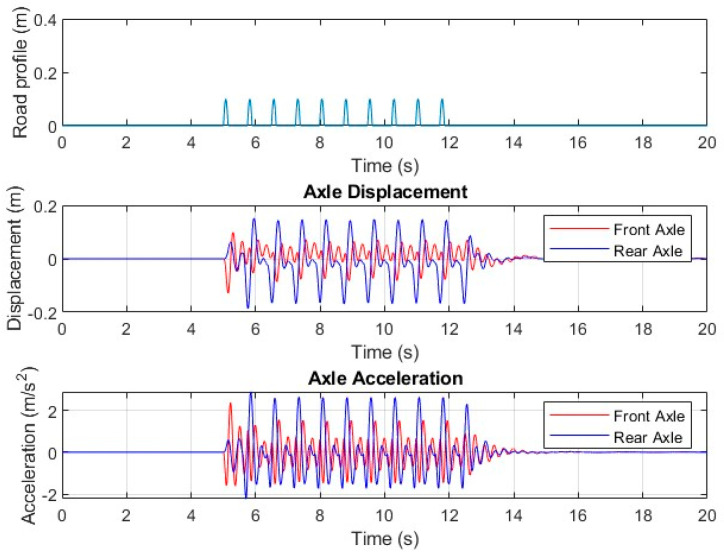
Response of a vehicle traveling over a road profile.

The above equations of motion were implemented in MATLAB (version 2022b) using the built-in solver *lsim*. A numerical example is presented here using the following parameters, and results are illustrated in Figure 7. It can be observed that the vehicle dynamics were readily described by the set of differential equations.

- *m_c_* = 3500 kg
- *m_f_* = *m_r_* = 500 kg
- *l_f_* = 1.5 m
- *l_r_* = 3.5 m
- *I =* 3000 kgm^2^
- *k_f_* = *k_r_* = 30 kN/m
- *k_tr_* = *k_tf_* = 200 kN/m
- *c_f_* = *c_r_* = 1500 Ns/m

## 3. Proof-of-Concept Road Tests and Prototype Development

### 3.1. Road Tests

**Figure 8:**
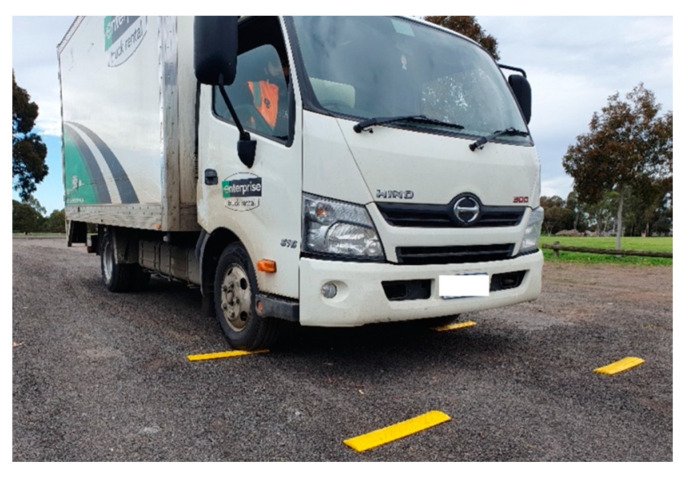
Test vehicle—3.5-ton truck.

**Figure 9:**
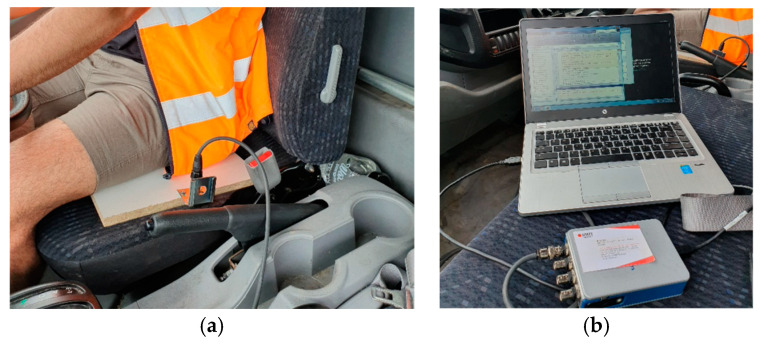
(**a**) Placement of accelerometer and (**b**) DAQ device.

**Figure 10:**
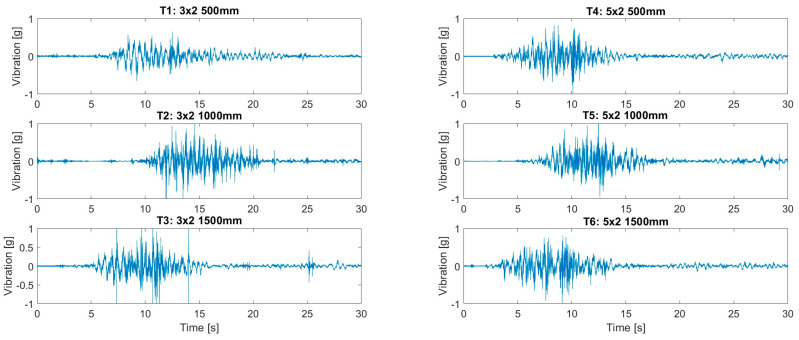
Measured driver seat vibrations.

To demonstrate the effects of rumble strips on the driver inside a truck cabin, road tests were conducted. Parameters of interest included vibrations experienced by the driver and the noise level in the cabin. It should be noted that the road test was conducted in a proof-of-concept manner rather than a comprehensive road test over a range of different vehicles and/or speeds. A 2-axle, 3.5-ton truck was used as a test vehicle, as shown in Figure 8. The road tests were conducted in a closed car park paved with gravel and dirt. Due to safety requirements, the speed of the truck was limited to 30 km/hr. The test truck had a 4.3 m clearance, and its cargo bay was empty. Plastic rumble strips measuring 500 mm × 100 mm × 15 mm (thickness) were secured by screws onto the road surface. Six rumble strip arrangements were tested, as listed in Table 1. Vibration of the driver seat and cabin noise level were measured. For instrumentation, an accelerometer (manufactured by PCB Piezotronics, model 603C01, sourced in Melbourne, Australia), sensitivity = 100 mV/g) was secured to a timber board on the driver’s seat in order to measure the vibration in the upright direction, as shown in Figure 9a. A National Instrument NI-9234 input module acquired the vibration data at 2048 Hz. A standard sound level meter was used to measure cabin noise. Measurement was made in a 30 s window passing over the rumble strips. The vibrations of the truck while it was accelerating and braking were excluded from the 30 s window. Each run was repeated three times to obtain averaged results. Results are summarized in Table 1 and vibration measurements are shown in Figure 10.

**Figure 11:**
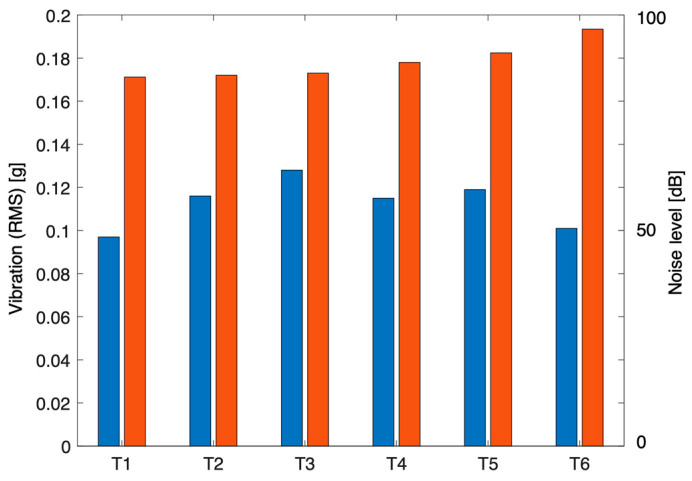
Road test results.

The vibration time histories clearly indicated that significant vibration was experienced by the driver due to the presence of rumble strips. The time span of high vibrations depended on the speed of the truck and the length of the rumble strips. The peak accelerations summarized in Table 1 are unfiltered values and may represent instantaneous vibrations that were measured. Instantaneously, the driver experienced vibration levels generally exceeding 1.0 g and, in T3, exceeding 3.0 g. The RMS values, on the other hand, are more representative values for comparison. It was observed that rumble strips arranged with 1500 mm spacings with three strips in a row generated the highest vibrations. The average RMS obtained was 0.113 g. However, the difference between different strip arrangements is insignificant. The noise level recorded in the cabin generally reached 90 dB in all runs. Comparing the cabin noise measurements showed insignificant differences between strip arrangements. RMS vibration and noise level are graphically compared in Figure 11.

**Figure 12:**
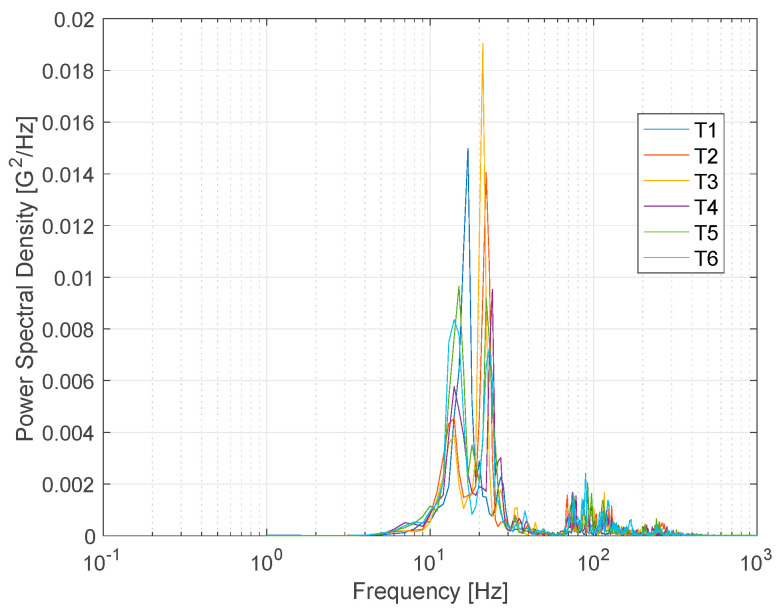
Power spectral density of measured vibration.

Vibration data obtained from road tests were analyzed in the frequency domain. Figure 12 shows the power spectral density estimated using Welch’s overlapped segment averaging estimator in MATLAB. It was observed that frequencies are concentrated in the band between approximately 12–27 Hz. This range is consistent with the “primary vehicle axles oscillation” found in a previous study on the vibration of a heavy vehicles over an expansion joint [30]. The driver’s position (Figure 8) was directly above the front axle of the test truck. Again, different rumble strip spacings and arrangements used in the test run did not result in significant changes in the frequency content experienced by the driver.

### 3.2. Prototype Development

**Figure 13:**
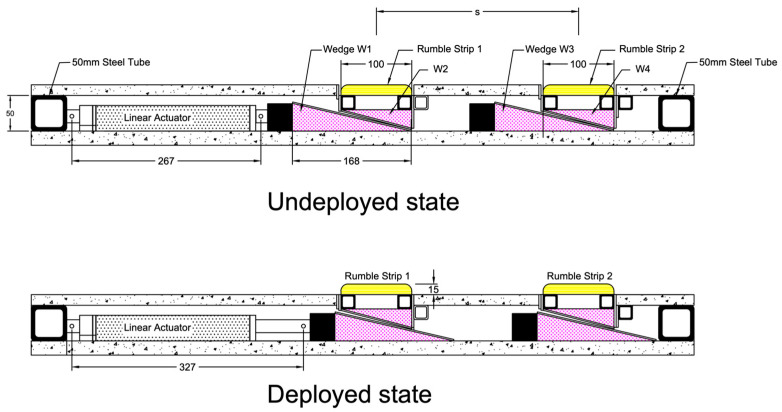
Smart rumble strip system prototype—section view.

**Figure 14:**
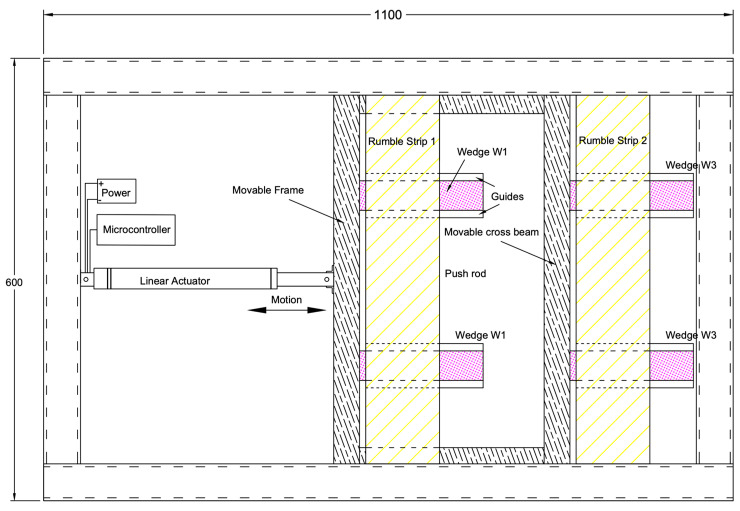
Smart rumble strip system prototype—plan view.

**Figure 15:**
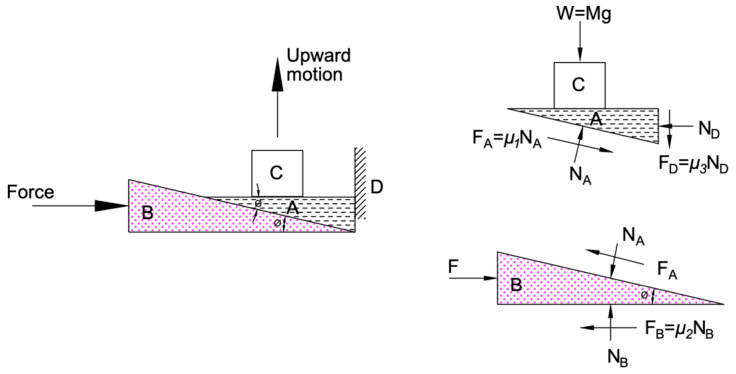
Wedge mechanism to lift rumble strips.

Under the physical constraints of a university laboratory, a prototype that consisted of two mechanized rumble strips was fabricated. The development of the prototype aimed to provide a proof-of-concept basis, with the prototype capturing most of the electronic and mechanical details required for actual implementation. The design principle was simple, keeping the number of moving parts minimal. Figure 13 and Figure 14 show a design drawing. The mechanized rumble strip system was fitted inside an enclosed frame of 1100 mm (length) × 600 mm (width) × 75 mm (thickness). A steel frame consisting of 50 mm square tubes formed a rectangular enclosure, which provided the structural support of the system and also housed the electrical/mechanical components. For a simple fabrication process, plywood was used for the bottom and top plate enclosures. For actual road implementation, the plywood should be replaced by steel plates to provide robustness. A major challenge in the design and fabrication was elevating the rumble strips while providing sufficient vertical strength. After several unsuccessful attempts, such as using an electric stepper motor and gear system or rumble strips supported on guide rails, the final design used a wedge concept to lift up the rumble strips. Figure 15 shows free-body diagrams of the wedge concept used. Object C represents the weight of a rumble strip, which rests on a wedge A. Force *F* drives wedge B underneath wedge A. While wedge A is restrained on the right by the boundary D, object C is lifted. Upon examining the free-body diagram, the driving force needs to overcome three frictional surfaces, with friction coefficients denoted by *μ*_1_ to *μ*_3_. The wedge concept provides a mechanical advantage in that a smaller driving force will be able to lift a heavier weight. Applying this concept in the mechanical rumble strips facilitated a single actuator to lift a series of rumble strips. Assuming the friction coefficients *μ*_1_ to *μ*_3_ = 0.3 and equals to 20 degrees, it can be shown that a mechanical advantage of approximately 3.4 is achieved.

**Figure 16:**
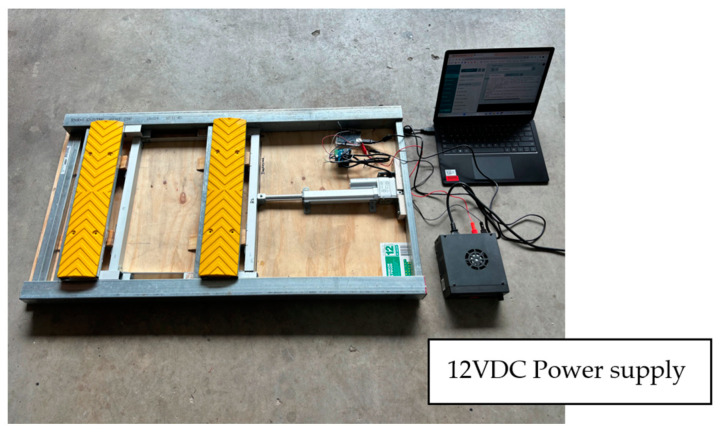
Smart rumble strip prototype.

Actuation was delivered by an electric linear actuator that pushed a movable frame forward and backward. Deploying and retracting the rumble strips were achieved using wedges. The actuator pushed the triangular wedges (W1 and W3) to elevate the rumble strips, which were attached to W2 and W4. When the actuator retracted, the rumble strips were lowered and a flat surface was restored. The friction between the wedge surface prevented damage to the actuator and other mechanical parts when a heavy wheel load was exerted on the strips. These figures show the method of mobilizing multiple rumble strips by a single actuator. Wedges W1 and W3 were attached to a movable frame. In principle, more rumble strips can be added to the system provided that the actuator is powerful enough to overcome the frictional forces. Figure 16 shows the completed prototype with the top cover removed to show the components inside. An optical feedback linear actuator manufactured by Firgelli Automation Australia (sourced in Melbourne, Australia) was used. The actuator has a 6-inch (152 mm) stroke.

**Figure 17:**
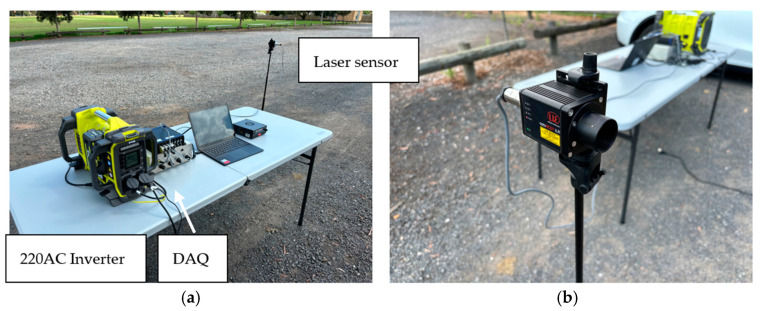
(**a**) Overview of setup and (**b**) laser distance measurement sensor.

The completed prototype system was verified in an outdoor and daylight environment, as shown in Figure 17a. The aim was to verify vehicle detection and the activation of the rumble strips in an outdoor environment. Vehicle detection was instrumented by a Micro-Epsilon laser displacement sensor (manufactured by Micro-Epsilon, model optoNCDT ILR 2250, sourced in Melbourne, Australia), as shown in Figure 17b. The sensor may measure the distance of a reflective surface for up to 150 m. The passing of a vehicle was detected as a momentary reduction in measured distance. The sensor was connected to a data acquisition device (DAQ) (manufactured by ME-Meßsysteme, model GSV-8, sourced in Melbourne, Australia) measuring amplifier and the sampling rate was set to 25 Hz. The GSV-8 unit was connected to a laptop computer. The distance signal was fed to a microcontroller (Arduino UNO with Atmel ATmega328 microcontroller, which operates at 16 MHz, sourced in Melbourne, Australia). Once a threshold was exceeded, the microcontroller signaled to extend the actuator and raise the rumble strips. In this prototype, the actuator operated at 12 VDC and required a separate power supply. To provide power to the actuator and sensing during field verification, a battery-powered, portable 220 V converter (manufactured by Ryobi, model name 36V Station Inverter, sourced in Melbourne, Australia) was used. A time delay was implemented in the codes of the microcontroller such that the rumble strips would be retracted 10 s after the over-height vehicle was detected. The field verification was successful when the mechanical rumble strips were deployed when an obstruction was detected by the laser distance sensor. The strips were retracted after a 10 s delay.

## 4. Discussion on Practical Implementation

The previous sections discuss the conceptual development of a smart rumble strip system and provided verification for a proof-of-concept prototype. For successful field application, the system will be required to fulfill several design criteria. They are divided into three categories and discussed herein:

Geometric requirements
  - The overall housing of the system is shallow to minimize excavation on existing pavement.
  - Adequate distance between the sensors and the smart rumble strips, allowing for the deployment to complete before the offending vehicle arrives. This is a function of design speed of the strip of the road.
Detection and control requirements
  - An over-height detection system, such as an infrared beam break sensor or a non-contact displacement sensor, is used to identify vehicles that exceed height restrictions. The detection system must operate in day and night conditions.
  - A microcontroller receives the signal from the sensor and triggers the deployment of rumble strips. The microcontroller must be contained in a robust housing within the smart rumble strip system to avoid the ingress of water or dust. The control unit may also be moved to a roadside housing.
Mechanical and electrical requirements
  - Rumble strips should be designed with sufficient strength to withstand design axle wheel loads in both deployed and undeployed states.
  - Stringent protection against water and dust ingress.
  - Efficient mechanical design is necessary to ensure a swift response with low power consumption.
  - Availability of electrical power supply.

Further road tests are envisaged to determine the vibrations and noise level generated by different numbers and lengths of rumble strips. Field trials are also necessary to review unforeseeable issues prior to wider application.

## 5. Conclusions

Collisions of over-height vehicles with low clearance bridges are common problems encountered worldwide. Minor collisions lead to damage to vehicles and the interruption of traffic; while more serious collisions lead to injuries, fatalities and damage to infrastructure. This paper presents a novel concept for mitigating such risk. The concept combines over-height detection and warning (OHD-W) systems with a mechanized rumble strip system. Over-height vehicle detection is carried out via beam break technology. Once an over-height event is detected, rumble strips are deployed by an actuator causing an uneven road surface. The strips will produce noise and noticeable vibrations to alert the driver to stop the vehicle. Unlike conventional OHD-W techniques, which only deliver visual warning signs or occasional warning sounds that drivers may still ignore, the proposed system produces a vigorous warning to drivers and decelerates the offending vehicle. The contribution of this paper includes the following:

- A conceptual framework of a smart, sensor-triggered mechanical rumble strip system was presented;
- The governing equations of a half car model traveling over a rumble strip road profile were reviewed and a numerical example was illustrated;
- Physical road tests were conducted using a 3.5-ton truck to quantify the cabin noise and vibration generated by different arrangements of rumble strips;
- A proof-of-concept model testbed with controlling software and mechanical design was tested and verified in an outdoor environment;
- Requirements for full-scale implementation were discussed.

This article demonstrates that the smart rumble strip system is simple to design and fabricate. The proposed event-triggered rumble strip system could significantly mitigate over-height vehicle collisions that cause significant disruption and injuries worldwide. A full-scale investigation is envisaged to convert this concept into a full engineering solution.

## Figures and Tables

**Table 1:** Summary of road test.

Test ID	Rumble Strip Arrangement	Spacing (mm)	Peak Acceleration (g)	Acceleration RMS (g)	Cabin Noise (dB)
T1	3 × 2	500	0.656	0.097	85.6
T2	3 × 2	1000	1.679	0.116	86.0
T3	3 × 2	1500	3.389	0.128	86.5
T4	5 × 2	500	1.020	0.115	89.0
T5	5 × 2	1000	1.312	0.119	91.2
T6	5 × 2	1500	1.052	0.101	96.7

## Data Availability

The raw data supporting the conclusions of this article will be made available by the authors on request.
